# GPCR Inhibitors Have Antiviral Properties against JC Polyomavirus Infection

**DOI:** 10.3390/v16101559

**Published:** 2024-09-30

**Authors:** Amanda L. Sandberg, Avery C. S. Bond, Lucas J. Bennett, Sophie E. Craig, David P. Winski, Lara C. Kirkby, Abby R. Kraemer, Kristina G. Kelly, Samuel T. Hess, Melissa S. Maginnis

**Affiliations:** 1 Department of Molecular and Biomedical Sciences, University of Maine, Orono, ME 04469, USA; amanda.sandberg@maine.edu (A.L.S.); avery.bond@maine.edu (A.C.S.B.); lucas.bennett@maine.edu (L.J.B.); sophie.craig@maine.edu (S.E.C.); lara.kirkby@maine.edu (L.C.K.); abby.kraemer@maine.edu (A.R.K.); kristina.kelly@maine.edu (K.G.K.); 2 Graduate School of Biomedical Science and Engineering, University of Maine, Orono, ME 04469, USA; samuel.hess@maine.edu; 3 Department of Physics & Astronomy, University of Maine, Orono, ME 04469, USA; david.p.winski@maine.edu

**Keywords:** JC polyomavirus, progressive multifocal leukoencephalopathy, PML, GPCR agonists/antagonists, cetirizine, paroxetine, 5-HT_2_Rs, β-arrestin, super-resolution microscopy

## Abstract

JC polyomavirus (JCPyV) infects the majority of the population and initially establishes a persistent but asymptomatic infection of the kidneys. In healthy individuals, the infection remains controlled by the host immune system, but for individuals experiencing prolonged immunosuppression, the infection can reactivate and spread to the brain, where it causes progressive multifocal leukoencephalopathy (PML), which is a fatal neurodegenerative disease. Currently, there are no approved therapies to treat PML, and affected individuals suffer rapid motor weakness and cognitive deterioration. To identify novel therapeutic treatments for JCPyV infection, receptor agonists/antagonists identified in a previously published drug screen were evaluated for their antiviral properties. Seven drugs were selected and validated using infectivity assays, and the mechanism of inhibition was further explored for G protein coupled receptor (GPCR)-associated inhibitors due to the role of the GPCR 5-hydroxytryptamine 2 receptors (5-HT_2_Rs) in JCPyV entry. The inhibitors cetirizine and paroxetine both reduced infection early in the JCPyV infectious cycle. Paroxetine specifically reduced viral internalization through altering the receptor density of 5-HT_2C_R, inhibiting β-arrestin recruitment to the receptor, and reducing MAPK signaling through ERK. These findings highlight the potential of receptor signaling and viral entry mechanisms as possible targets for antiviral drug development. Further, this research suggests that FDA-approved receptor agonists/antagonists currently used to treat other medical conditions could be repurposed into antivirals for the possible treatment of JCPyV infection and the fatal disease PML.

## 1. Introduction

JC polyomavirus (JCPyV), a small non-enveloped double-stranded DNA virus, infects 50–80% of the population [1,2]. The virus is shed in urine and is transmitted via the fecal–oral route. Infection commonly occurs during early childhood through adulthood and results in a lifelong infection in the kidneys [3,4]. In healthy individuals, the initial infection is maintained at low levels and presents asymptomatically. However, during severe prolonged immunosuppression, JCPyV can spread from the kidneys into the central nervous system, where it results in a lytic infection of the glial cells in the brain, specifically astrocytes and oligodendrocytes [5,6]. JCPyV infection of the brain leads to the disease progressive multifocal leukoencephalopathy (PML), which is characterized by rapid neural demyelination and lesion formation. Symptoms of PML include impaired sensory function, paralysis, and the deterioration of cognitive abilities [7]. Early detection and effective treatments of the underlying immunosuppression have increased the survival rate of PML, but despite these efforts, the disease outcome is often fatal: sometimes as rapid as within a year of symptom onset [5,8]. Viral spread and the development of PML is most common in individuals with uncontrolled human immunodeficiency virus (HIV) infection or those undergoing prolonged immunomodulatory treatments for cancer, organ transplant or immune-mediated diseases such as multiple sclerosis (MS) [9].

Currently, there are no approved targeted treatments for JCPyV infection or PML, and clinicians are limited to treating the underlying immunosuppression by ceasing the use of immunomodulatory drugs or implementing highly active antiretroviral therapy (HAART) for HIV management [5,10]. A significant risk of immune system rescue is the development of PML immune reconstitution inflammatory syndrome (IRIS), which is a disease with substantial morbidity that sometimes leads to worsening of the neurological deficits caused by PML [11,12]. Direct antiviral therapies most commonly targeting viral entry or replication have been evaluated with limited success. Nucleoside analogues, cytarabine and cidofovir, topoisomerase inhibitor topotecan and serotonin receptor antagonist mirtazapine have shown promising reductions of infection in vitro but lacked clinical benefit or relevance in clinical trials [5,13,14,15,16]. The absence of approved and effective treatments for PML emphasizes the critical need for additional research exploring the infectious cycle of JCPyV to identify novel therapeutic targets and antiviral therapies.

JCPyV infection can occur through both receptor-independent entry through extracellular vesicles and receptor-mediated entry [17]. To initiate infection through receptor-mediated entry, the external component of the JCPyV capsid, viral protein (VP) 1, interacts with alpha2,6-sialic acid containing lactoseries tetrasaccharide (LST-c) and glycosaminoglycans (GAGs) on the host cell membrane [18,19]. Following attachment, the virus enters the cell through clathrin-mediated endocytosis using the G protein-coupled receptor (GPCR) type 2 serotonin receptor, 5-hydroxytryptamine receptor (5-HT_2_R) subtypes A, B and C [20,21,22]. Super-resolution microscopy studies have shown that JCPyV localizes with 5-HT_2_Rs during viral entry and that viral particles induce clustering of the 5-HT_2_Rs at times consistent with entry [23]. For proper viral endocytosis to occur, the 5-HT_2_Rs must be bound by scaffolding protein β-arrestin. The G protein-coupled receptor kinase 2 (GRK2) is also required for infection, presumably by initiating β-arrestin recruitment to the receptor [24,25,26,27]. Activation of the 5-HT_2_R also leads to the recruitment of additional endocytic proteins clathrin and adaptor protein (AP2), resulting in viral entry into clathrin-coated pits [24]. Following clathrin-mediated endocytosis, the virus traffics in endosomes to the ER and lastly to the nucleus, where the viral genome is transcribed and replicated [28]. β-arrestin recruitment by the 5-HT_2_Rs has also been suggested to trigger the host mitogen-activated protein kinase (MAPK) cascade, which is a signaling pathway necessary for JCPyV to complete the infectious cycle [29,30]. The MAPK cascade terminates in the phosphorylation of extracellular signal-regulated kinase (ERK), which recruits and activates host transcription factors to drive productive viral infection [30,31,32]. Inhibitors of 5-HT_2_Rs, clathrin-mediated endocytosis, and the MAPK cascade significantly reduce JCPyV infection, suggesting that viral entry and GPCR signaling are promising therapeutic targets [21,22,23,30,33].

Additional support for viral entry as a promising therapeutic target was discovered in a large-scale high-throughput drug screen of the National Institute of Health Clinical Collection performed by Bond et al. [34]. In this study, the largest portion of drugs identified as hits that reduced JCPyV infection in vitro (36%) were characterized as receptor agonists/antagonists, particularly of GPCRs, making this group of considerable interest due to the known involvement of GPCRs and GPCR signaling in the JCPyV infectious cycle [22,30,31,34]. Further, GPCR signaling is a conserved mechanism of infection across viral families, as GPCR antagonists have been shown to reduce the infection of flaviviruses, influenza virus, and SARS-CoV-2, among others [26].

This study characterized potential antiviral treatments against JCPyV by validating seven receptor-specific inhibitors identified as hits in the high-throughput drug screen published by Bond et al. [34]. Hits that were approved by the Food and Drug Administration (FDA) with an average target receptor gene count higher than zero in SVGA cells [35] were evaluated, and drugs reducing infection were further assessed for their mechanistic action. Two GPCR-associated inhibitors, cetirizine and paroxetine, significantly reduce JCPyV infection across immortalized and primary cell types at early time points in the infectious cycle correlating with viral entry and GPCR signaling. The results described herein suggest viral entry and GPCR signaling as possible therapeutic targets to inhibit JCPyV infection and the potential of 5-HT_2_R- and GPCR-related antagonists to be repurposed and used as antiviral therapeutics.

## 2. Materials and Methods

### 2.1. Cells and Viruses

SVGA cells were maintained in complete minimum essential medium (MEM) (Corning, Corning, NY, USA) containing 10% fetal bovine serum (FBS) (Bio-techne, Minneapolis, MN, USA) 1% penicillin–streptomycin (P/S) (Corning) and 0.2% Plasmocin (InvivoGen, San Diego, CA, USA). Cells were passaged 2–3× weekly upon 90–100% confluency. Human embryonic kidney cells (HEK293A) stably expressing 5-HT_2C_R tagged with YFP (HEK-2C-YFP) [22] or Dendra2 (HEK-2C-Dendra2) [23] were maintained in Dulbecco’s modified Eagle’s medium containing (DMEM) (Corning) 10% FBS, 1% P/S, and 0.2% Plasmocin. To sustain receptor expression, cells were maintained in 1% G418 (MP Biomedicals, Irvine, CA, USA). Cells were passaged 2–3× weekly upon 90–100% confluency, and media was replaced every 48 h (h). Normal human astrocytes (NHAs) were maintained in complete astrocyte medium (CAM) with 2% FBS, 1% P/S, and 1% astrocyte growth supplement (ScienCell, Carlsbad, CA, USA). Cells were passaged upon 90% confluency in accordance with ScienCell distributer protocol, and media was replaced every 48–72 h. Renal proximal tubule epithelial cells (RPTECs) were maintained in complete renal epithelial growth medium (REGM) (ATCC, Gaithersburg, MD, USA) with an added epithelial cell growth kit (ATCC) and 1% P/S. Cells were passaged upon 90% confluency in accordance with ATCC protocol, and media was replaced every 48 h. All cell lines were propagated in a humidified incubator at 37 °C with 5% CO_2_ and tested for mycoplasma every 6 months. SVGA cells and HEK-2C cells were generously provided by the Atwood Laboratory (Brown University, Providence, RI, USA), while RPTECs and NHAs were obtained from ATCC and ScienCell, respectively.

JCPyV strain Mad-1/SVEΔ was generated and propagated as described in [28,36]. All experiments, except when purified labeled virus is indicated, were performed using a crude supernatant virus stock titered by focus-forming unit (FFU) infectivity assay in SVGA cells. JCPyV was purified as described in [28], and pure JCPyV was labeled with Alexa Flour 647 as described in [18].

### 2.2. Agonists/Antagonists and Antibodies

All inhibitors used had a purity of at least 98% and include telmisartan (Selleckchem, Houston, TX, USA), paroxetine (Abcam, Cambridge, MA, USA), cetirizine (Selleckchem), pergolide (Medchem Express), pilocarpine (Selleckchem), pindolol (Sigma Aldrich, St. Luois, MO, USA), and rizatriptan (Selleckchem). All drugs were resuspended in DMSO (Tocris Bioscience, Minneapolis, MN, USA), and DMSO was also used as a volume-specific vehicle control. Figures or figure legends list the concentrations used for each inhibitor.

The primary antibodies used in FFU and ICW assays include PAB962, a monoclonal antibody (mAb) derived from a hybridoma supernatant for the detection of the JCPyV large TAg protein graciously provided by the Tevethia Laboratory (Penn State University, Hershey, PA, USA) [37], and an antibody against phosphorylated ERK (pERK) (Cell Signaling Technology, Danvers, MA, USA). Secondary antibodies used include polyclonal goat anti-mouse and goat anti-rabbit Alexa Fluor 488 and 594 antibodies (Thermo Fisher, Waltham, MA, USA) and LI-COR 800 goat anti-mouse and anti-rabbit antibodies (LI-COR, Lincoln, NE, USA). DAPI (Thermo Fisher) was used to stain cell nuclei in FFU assays, and CellTag 700 (LI-COR) was used as a cell count normalization stain for In-Cell Western assays. Antibodies used for staining after pulldown assay and Western blot include primary antibodies against β-arrestin and GFP (Cell Signaling Technology) as well as a LI-COR 800 anti-rabbit secondary antibody (LI-COR). Entry assays quantified using confocal microscopy were stained using a primary antibody against pan-cadherin (Abcam) and a polyclonal goat anti-mouse Alexa Fluor 488 secondary antibody (Thermo Fisher).

### 2.3. Cell Viability Assay

The MTS assay (G3581) (Promega, Madison, WI, USA) was used according to the manufacturer’s instruction to determine the cell viability for each cell type after the treatment of specified inhibitor concentrations. To mimic infectivity assay protocols, cells were plated to 70% confluency and pre-treated with drug or DMSO volume control at 37 °C for 1 h. Cells were then mock infected with appropriate media alone and incubated with an inhibitor or DMSO control for 48 h (SVGA and NHAs) or 72 h (RPTECs). MTS reagent was added for 1 h at 37 °C before absorbance was measured at 490 nm using an Agilent BioTek Cytation 5 Imaging Reader (Agilent Technologies, Santa Clara, CA, USA). Drug concentrations maintaining >80% cell viability in comparison to the appropriate DMSO control were deemed to not induce significant toxicity and were considered usable concentrations. Experiments were performed in triplicate wells for at least 3 replicates.

### 2.4. JCPyV Infection

SVGA, HEK-2C, RPTEC, and NHA cells were plated to 70% confluency in 96-well plates in MEM, DMEM, REGM, CAM or MEM, respectively. The inhibitors and DMSO volume control were diluted in appropriate media to concentrations indicated in figures and incubated at 37 °C for 1 h. Cells were infected with JCPyV at a multiplicity of infection (MOI) of 1 FFU/cell (SVGAs and HEK-2Cs), 3 FFU/cell (RPTECs), or 2 or 3 FFU/cell (NHAs) in the absence of drug at 37 °C for 1 or 2 h. Infections were fed with 100 uL/well of drug- or DMSO-containing media at appropriate concentrations and incubated at 37 °C for 48 h (SVGAs, HEK-2Cs, and NHAs) or 72 h (RPTECs). Cells were then fixed in 4% PFA at RT for 10 min directly in media, media was removed, and 4% PFA was added for another 10 min without media prior to processing via FFU or In Cell Western (ICW) assays.

### 2.5. FFU Infectivity Assay

Fixed cells were washed in 0.1% PBS-Tween (PBS-T) 3× for 5 min each before staining. Cells were permeabilized with 1% Triton X-100 in PBS at room temp (RT) for 15 min and blocked with 10% goat serum in PBS at RT for 1 h with gentle rocking. JCPyV TAg-specific primary antibody PAB962 (1:5) in PBS was added to the cells at RT for 1 h. Cells were washed 3× for 5 min each with PBS-T and then incubated with a polyclonal goat anti-mouse Alexa Flour 594 or 488 secondary antibody (1:1000) in PBS at RT for 1 h. Again, cells were washed 3× with PBS-T for 5 min each, and cell nuclei were stained with DAPI (1:1000) in PBS at RT for 5 min. PBS-T was used to wash 2×, and PBS was added for storage at 4 °C. All infections were performed in triplicate wells for at least 3 biological replicates.

Infected and stained cells were visualized and quantified using epifluorescence microscopy on a Nikon Eclipse Ti microscope (Micro Video Instruments Inc., Avon, MA, USA). The percent infection was quantified by dividing TAg-positive cells by the number of total cells visualized by DAPI and multiplying by 100. Quantification was performed at 10× or 20× magnification for 5 fields of view (FOV) per well. DAPI-positive cells were counted using an automated binary algorithm in the Nikon NIS Elements Basic Research software (versions 4.50.00 and 6.02.01) separating cells by intensity, diameter, and circularity, while TAg-positive cells were counted manually [25,34].

### 2.6. In-Cell-Western Assay

Fixed cells were washed with PBS-T 3× for 5 min each and permeabilized with TX-100 in PBS at RT for 15 min. Cells were then blocked with TBS Odyssey Blocking Buffer (LI-COR) at RT for 1 h before being incubated with primary antibody specific for JCPyV TAg (PAB962) (1:5) or pERK (1:750) in TBS Odyssey Blocking Buffer at 4 °C overnight (O/N) with gentle rocking. Cells were washed 3× in PBS-T 5 min each and then incubated with LI-COR 800 secondary anti-mouse or anti-rabbit antibody (1:10,000) and CellTag 700 (1:500) at RT for 1 h. Lastly, cells were washed 3× with PBS-T.

Cells were washed with PBS, and liquid was removed before scanning at 700 and 800 nm channel intensities using a LI-COR CLx Infrared Imaging system. The scanner was set to 42 μm resolution, medium quality, and 3.0 mm focus offset [32]. Image studio software was used to align channels after scanning, and the ICW module was used to outline each well. The intensity values inside the wells for the 700 and 800 channels were recorded, and infection was quantified by dividing the 800-channel intensity value by the 700-channel intensity value and multiplying by 100. Finally, values were normalized to the appropriate control [34].

### 2.7. Time of Addition Assay

SVGA cells were plated to 70% confluency in 96-well plates using complete MEM. Treatment with inhibitors was initiated either 1 h before infection (pre-treatment), during infection (0 hpi), or at indicated time points after infection (2, 4, 6, 12 or 24 hpi). For all treatment conditions, drugs were diluted in 10% MEM and incubated at 37 °C. Infection with JCPyV was performed in the presence of drug at 37 °C unless otherwise indicated, and infection was fed with 10% MEM containing drug or 10% MEM alone. At 48 hpi, cells were fixed with 4% PFA at RT for 10 min directly in media, media was removed, and 4% PFA (without media) was added for another 10 min. Cells were stained for JCPyV TAg (1:5) and analyzed via ICW assay. Experiments were performed in triplicate for 3 biological replicates.

### 2.8. pERK Signaling Assay

SVGA cells were plated to 90% confluence in 96-well plates using complete MEM. Inhibitors were diluted to concentrations indicated in figures in 10% MEM and incubated at 37 °C for 1 h. Cells were then pre-chilled at 4 °C for 15 min before being mock infected with MEM or infected with JCPyV in the absence of drugs at 4 °C for 1 h. Infections were fed with 10% MEM containing appropriate drug concentrations at 37 °C for 15 min and then fixed in 4% PFA at RT for 10 min directly in media, media was removed, and 4% PFA (without media) was added for another 10 min. Cells were stained for pERK (1:750) and CellTag (1:500) and analyzed via ICW assay. Experiments were performed in triplicate for 3 biological replicates.

### 2.9. Attachment Assay by Flow Cytometry

SVGA or HEK-2C cells were plated to 100% confluency in 12-well plates and treated with DMSO or drug at 37 °C for 1 h. Cells were washed with PBS and then incubated with Cellstripper (Corning) at 37 °C for 15 min to detach cells from the plate. Detached cells were pelleted at 452× *g* at 4 °C for 5.5 min and washed with PBS. JCPyV labeled with Alexa Fluor 647 (JCPyV-647) in phenol red free MEM was added to cells and incubated on ice for 1 h with agitation every 15 min. Cells were washed with PBS, pelleted by centrifugation, then fixed in 4% PFA on ice for 10 min. Each sample was resuspended in PBS and analyzed for viral attachment by flow cytometry using an LSRII system (BD Biosciences) with a 650 nm AP-C laser line. At least 10,000 events were recorded per sample, and data analyses were performed using BD FACSDiva (version 6.2) and FlowJo software (version 10.10). Gates were generated to exclude dead and complex cells.

### 2.10. Entry Assay by Confocal Microscopy

SVGA or HEK-2C-YFP cells were plated to 70% confluence in 96-well glass-bottom plates (CellVis number 1.5, CellVis, Sunnvale, CA, USA). Cells were pre-treated with drug or DMSO control in 10% MEM (SVGA) or 10% DMEM (HEK-2C-YFP cells) at 37 °C for 1 h and then pre-chilled at 4 °C for 45 min. JCPyV labeled with Alexa Fluor 647 [17] (MOI = 4 FFU/cell) was added at 4 °C for 1 h, and cells were then fed with pre-warmed media containing drug or DMSO at 37 °C for 2 h. Fixation was completed by adding 4% PFA at RT for 10 min with media present, removing the media, and adding 4% PFA (without media) for another 10 min. After fixation, SVGA cells were washed with PBS 3× and incubated in block buffer (2% goat serum, 0.2% TX-100 and 0.1% BSA) at RT for 1 h while rocking. A primary antibody against pan-cadherin (1:75) in block buffer was added to the cells and incubated at 4 °C O/N while rocking. Cells were washed 3× with PBS and incubated with anti-mouse 488 secondary antibody (1:1000) at RT for 1 h while rocking. Another 3 washes with PBS were performed, and DAPI (1:1000) was added at RT for 10 min while rocking. After fixation, HEK-2C-YFP cells were washed 3× with PBS, stained with DAPI (1:1000) at RT for 10 min while rocking, and then washed 3× with PBS. Plates were stored in PBS until visualization. Samples were visualized at 63× magnification (oil immersion) using a Zeiss LSM 980 Confocal Microscope with Airyscan 2 and the Zen Blue 3.8 software. Diodes of 405 nm, 488 nm and 639 nm were used to acquire images, and cross-sections of at least 30 cells per sample were analyzed. Images were analyzed in ImageJ for viral internalization. HEK-2C-YFP cell images were subjected to a median filter in ImageJ to remove background noise. Region of interest (ROIs) were defined using the polygon selection tool to exclude the plasma membrane in either the 488 channel or differential interference contrast (DIC) images [23]. Viral internalization was measured by comparing relative fluorescence units per cell for background corrected samples. Experiments were performed for 3 biological replicates, and graphs represent at least 90 cells per treatment.

### 2.11. Serotonin Receptor Pulldown Assay

HEK-2C cells expressing 5-HT_2C_R tagged with YFP were plated to 90% in 6-well plates and pre-treated with paroxetine or DMSO at 37 °C for 1 h. Plates were then pre-chilled at 4 °C for 45 min and incubated with either JCPyV (MOI = 3 FFU/cell) or 5-HT [200 μM] in 10% DMEM at 4 °C for 1 h. Pre-warmed paroxetine/DMSO-containing DMEM was added to each well at 37 °C for 15 min before removal by scraping. Detached cells were pelleted at 414× *g* at 4 °C for 10 min and resuspended in lysis buffer (10 mM Tris HCl, 150 mM NaCl, 0.5 mM EDTA, and 0.5% Triton X-100 [pH 7.5]) supplemented with phosphatase (Sigma-Aldrich) and protease (Sigma-Aldrich) inhibitors on ice for 30 min with vigorous mixing every 10 min. Cells were pelleted at 21,130× *g* at 4 °C for 10 min, while GFP-Trap magnetic agarose beads were washed 3× with wash buffer (10 mM Tris HCl, 150 mM NaCl, 0.5 mM EDTA, and 0.5% Triton X-100 [pH 7.5]) and separated using a magnetic tube rack. The sample supernatant was mixed with the beads and incubated at RT for 2 h while rotating. Beads were washed 3× before being transferred to a new tube in 50 μL buffer. Samples were mixed 1:1 with Laemmli sample buffer (BioRad, Hercules, CA, USA) and boiled at 95 °C for 10 min. Samples were processed by SDS page using a 10% TGX mini gel (BioRad), and proteins were transferred to a nitrocellulose membrane (BioRad) at 2.5 amps (25 V) for 3 min using a BioRad Semidry Transblot unit. Membranes were equilibrated in TBS for 5 min followed by blocking using Odyssey blocking buffer (LI-COR) at RT for 1 h while rocking. TBS-T was used to wash membranes 3× before incubating with a β-arrestin 1/2 primary monoclonal antibody (1:500) in block buffer at 4 °C O/N. Membranes were washed 3× and incubated with LI-COR 800 anti-rabbit secondary antibody (1:10,000) in block buffer at RT for 1 h. Another TBS wash was completed before membranes were imaged on a LI-COR Odyssey CLx system using the 800 channel. Membranes were stripped of antibodies using NewBlot stripping buffer (LI-COR) according to manufacturer’s instructions and then re-probed with an anti-GFP primary monoclonal antibody (1:1000) to detect 5-HT_2_R-YFP at 4 °C O/N. Blots were then washed, incubated with LI-COR 800 anti-rabbit secondary antibody for the detection of 5-HT_2_R-YFP, and membranes were washed and imaged as previously described. ImageJ was used to determine percentage of bound β-arrestin by comparing the background subtracted mean signal of each β-arrestin band to the corresponding receptor protein band. Within each sample, the β-arrestin band was normalized to 5-HT_2_ expression, and results were compared across paroxetine- or DMSO-treated samples challenged with either 5-HT or JCPyV.

### 2.12. Receptor Cluster Analysis by Super-Resolution Microscopy

HEK-2C-Dendra2 cells were plated to 50% confluency in 96-well glass-bottom plates (CellVis) using phenol-free DMEM supplemented with 4 mM glucose, 10% FBS, and 1% G418 (Corning). Cells were treated with paroxetine or DMSO (vehicle) at 37 °C for 1 h and then pre-chilled at 4 °C for 1 h. Cells were incubated with JCPyV-647 at 4 °C for 1 h and then fed with pre-warmed media containing drug or DMSO for 5 or 15 min. Cells were fixed with 4% PFA for 10 min and washed 2× with PBS. Samples were stored in PBS until imaging [23].

Imaging was completed utilizing a custom fluorescence photoactivation localization (FPALM) microscope [38]; an Olympus IX71 inverted microscope with a 60X objective, 2× telescoping lens in the detection path, and an Andor iXon + electron-multiplying charge-coupled device for detection. Samples were imaged in GLOX buffer (10% *w*/*v* glucose, 10 ug/mL Catalase, 50 ug/mL Glucose OXidase, 1% beta-mercaptoethanol, and 10 mM Tris pH 7.4) to induce Alexafluor-647 blinking for directed stochastic optical reconstruction microscopy (dSTORM) [39,40]. Dendra2 activation and readout was achieved utilizing 405 nm and 558 nm lasers, respectively. Alexa Fluor-647 readout was achieved utilizing a 638 nm laser. Prior to beam splitting, two 405/488/561/635 notch filters (Semrock, Rochester, NY, USA) and a 561-notch filter (Semrock) located in the microscope turret were utilized to filter out the laser background. A DiO 405/488/561/635 multiband dichroic mirror (Semrock) also in the microscope turret was used to reflect lasers into the back aperture of the objective. Fluorescence collected by the objective passes through a tube lens and into a 2× telescope. After the telescope, the fluorescence reaches a FF580-FDi01 dichroic mirror (Semrock) that splits the light into the transmitted (red) and reflected (green) channels. The red channel has a 664 nm RazorEdge LP filter (Semrock), while the green channel has a 580/40 BrightLine filter (Semrock). A total of 10,000 images per cell were captured at a frame rate of 30 Hz and an electron multiplication gain of 200 [23].

Images were localized, concatenated, and analyzed (and rendered) utilizing custom MATLAB scripts [23,41]. Briefly, images were background subtracted utilizing a temporal median filter, and thresholds for blinking were set by sample. An α-histogram of relative fluorescence in both the red and green channels was utilized to select for Dendra2 and Alexaflour-647 localizations [41]. Dendra2 was identified as α < 0.7, and AlexaFluor-647 was identified as α > 0.83. Images from single cells were then concatenated, drift corrected, and duplicate pixels were removed [42]. Clusters were identified using a single-linkage cluster analysis (SCLA) algorithm [43]. Clusters were defined as imaged areas with a density of at least three times the cell-averaged density (units of localizations per unit area), a radius of at least 50 nm, and a minimum number of 10 localizations. Cluster properties were then analyzed for area, density, and perimeter, or radial distribution function (RDF) analysis was performed on each cluster, averaged over all clusters within a given cell, and then further analyzed or averaged [44]. Cluster density is represented by the average cluster density per cell. RDF functions show the average density at distance X from the cluster center. RDF functions are averages of all clusters found, and they are not differentiated by cell or replicate.

### 2.13. Statistical Analysis

Two-sample Student’s *t*-tests were performed using Microsoft Excel (Version 16.89.1) to determine statistical significance. Mean values of triplicate samples were compared assuming unequal variance. Variation in a given population was determined by calculating the standard error of the mean (SEM) by dividing the standard deviation of a population by the square root of the sample size.

Statistical differences in cluster densities were determined by a Wilcoxon signed-rank test, assuming unequal variance in population distributions due to the bimodal structure of population densities. RDF statistical analysis compared densities as a function of radius r measured from the cluster center (r = 0) out to r = 500 nm with a spacing of 10 nm. Statistical significance was quantified using a Wilcoxon signed-rank test with a Bonferroni–Holm correction.

## 3. Results

### 3.1. Hits Identified in High-Throughput Screen Target GPCRs

In an effort to identify and characterize antiviral therapeutics that could be used to treat or prevent JCPyV infection and the resulting brain disease PML, drugs identified as receptor agonist/antagonist hits in the high-throughput drug screen published by Bond et al. [34] were evaluated. Seven out of fifteen hits in this category were selected for further validation due to FDA approval status and targeting a GPCR that is expressed in the model cell line [37]. The drugs included serotonin receptor-related drugs paroxetine and rizatriptan and non-serotonin receptor-related drugs telmisartan, cetirizine, pergolide, pilocarpine, and pindolol (Table 1). These drugs are inhibitors of several different GPCRs, which are common pathways usurped by many viruses during the infectious cycle [26]. The hits were further selected based on having an average target receptor gene expression above zero counts in SVGA cells as reported in RNA sequencing data from our lab [45]. Focusing on already FDA-approved therapeutics provides the opportunity to repurpose effective drugs into antivirals for JCPyV and PML, which would prove faster than non-approved therapeutics.

### 3.2. Cell Viability of Immortalized and Primary Cell Lines

Cytotoxicity profiles were established for each of the drugs selected for further validation (Table 2). The cell viability of SVGA cells, NHA cells, and RPTE cells was determined at applicable time points upon drug treatment with a range of concentrations to establish appropriate working concentrations for each drug. Triplicate samples of at least three biological replicates were averaged for each concentration, and concentrations resulting in percent cell viability of 80% or higher were included in future validation experiments. Significantly toxic concentrations resulting in percent cell viability <80% were not included in future validation experiments.

### 3.3. Paroxetine and Cetirizine Reduce JCPyV Infection

**Figure 1:**
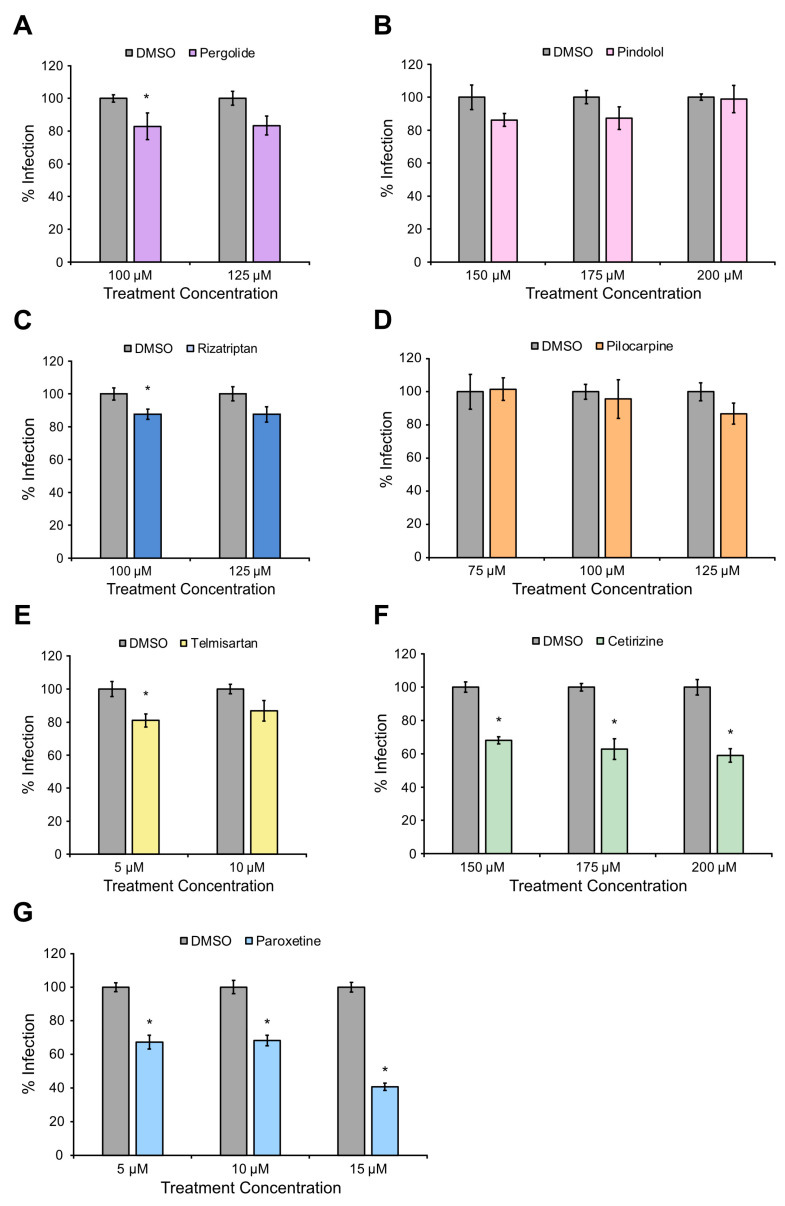
Cetirizine and paroxetine significantly reduce JCPyV infection in glial cells. SVGA cells were pre-treated with either drug or DMSO at 37 °C for (**A**–**D**,**F**,**G**) 1 h or (**E**) 2 h and then infected with JCPyV (MOI = 1 FFU/cell) in the absence of drug at 37 °C for 1 h. Cells were fed with media containing the drug or DMSO control and incubated at 37 °C for 48 h. Cells were fixed and stained for viral TAg and DAPI (total cells). FFU assay was used to quantify infection of 5 FOV/well at 20×, and percent infection was determined by dividing the number of TAg+ cells by the total number of cells and normalizing to 100%. Graphs represent 3 biological replicates performed in triplicate with error bars representing SEM. Statistical significance was determined using Student’s *t*-test. *, *p* < 0.05.

To validate the results from the high-throughput drug screen and identify candidates for mechanistic characterization, the effects of the therapeutics in Table 1 on JCPyV infection were explored using FFU assays. SVGA cells were pre-treated with each drug or DMSO control, infected with JCPyV, and infection was quantified using indirect immunofluorescence and epifluorescence microscopy. The results demonstrated that non-serotonin receptor-related drugs telmisartan, pindolol, pergolide, and pilocarpine did not reduce infection or resulted in only a modest reduction deemed biologically and clinically insignificant (Figure 1A,B,D,E). A similar result was observed for rizatriptan, which is a 5-HTR agonist (Figure 1C). Due to the lack of significant reduction in infection, the aforementioned drugs were not selected for further analysis. However, cetirizine (histamine H1 receptor antagonist) and paroxetine (serotonin reuptake transporter and GRK2 inhibitor) significantly reduced JCPyV infection by 41% and 59%, respectively, at the highest concentrations (Figure 1F,G). Cetirizine and paroxetine were selected for further study to identify how they function to reduce JCPyV infection.

### 3.4. Paroxetine and Cetirizine Reduce JCPyV Infection in Primary Kidney Cells and Astrocytes

**Figure 2:**
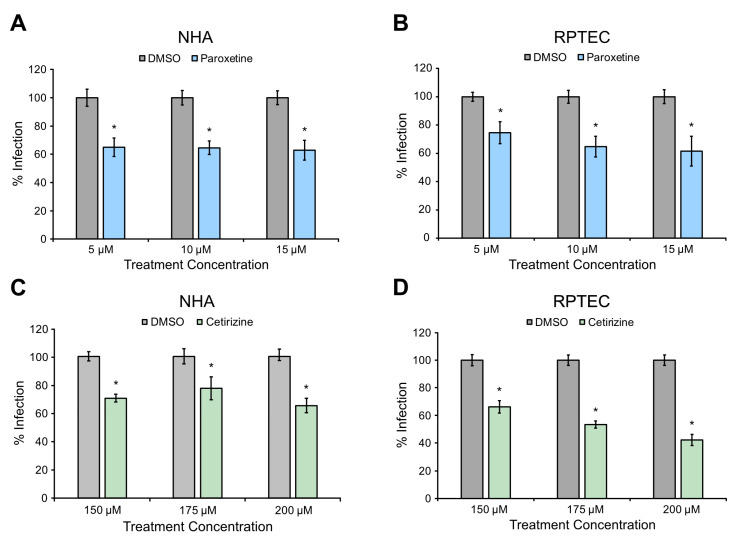
Paroxetine and cetirizine reduce JCPyV infection in primary glial and kidney cells. (**A**,**C**) NHA cells or (**B**,**D**) RPTECs were pre-treated with (**A**,**B**) paroxetine or (**C**,**D**) cetirizine at 37 °C for 1 h and infected with JCPyV ((**A**): MOI = 2 FFU/cell, (**B**–**D**): MOI = 3 FFU/cell) in the absence of drug at 37 °C for (**A**,**B**,**D**) 1 h or (**C**) 2 h. Cells were incubated in media containing DMSO or drug at 37 °C for (**A**,**C**) 48 h or (**B**,**D**) 72 h before being fixed. Cells were stained for TAg and DAPI, and FFU assay was performed to quantify infection of 5 FOV/well at (**B**,**D**) 20× or 10× (**A**,**C**). Percent infection was calculated by dividing the number of TAg+ cells by DAPI+ cells and normalized to 100%. Three biological replicates are presented in each graph, and error bars represent SEM. Statistical significance was determined using Student’s *t*-test. *, *p* < 0.05.

JCPyV infection is only permissive in human hosts, and currently, there is a lack of tractable animal models for infection. Possible models include primary cells, which compared to immortalized cells more accurately model the cells in a human host. In this study, primary cells of both the brain (normal human astrocytes (NHAs)) and the kidneys (renal proximal tubule epithelial cells (RPTECs)) were used to model infection. Primary cells were treated with cetirizine or paroxetine, and FFU assays were performed as described above. The findings show that paroxetine reduced infection up to 38% in both NHAs and RPTECs (Figure 2A,B), while cetirizine reduced infection up to 34% in NHAs and 58% in RPTECs. The reduction observed in primary cells is generally comparable to what was observed in immortalized SVGA cells.

### 3.5. Cetirizine Reduces Infection Early in the Infectious Cycle

**Figure 3:**
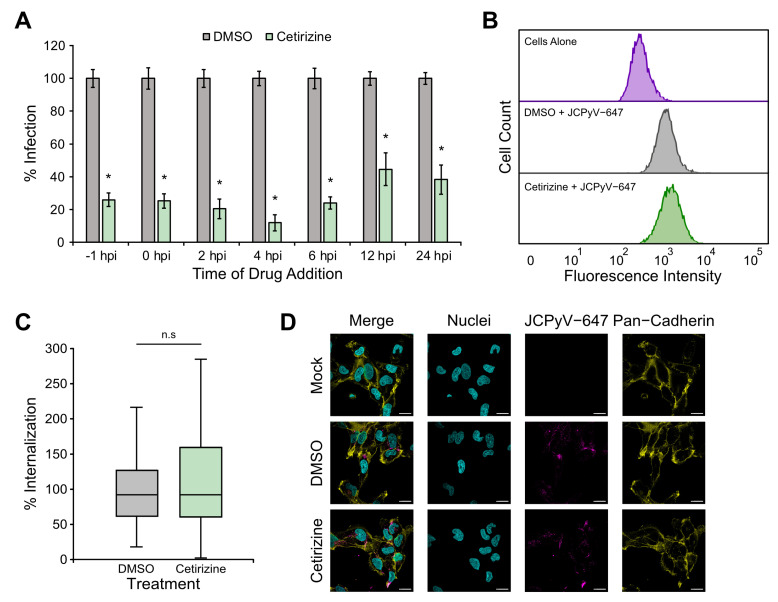
Cetirizine reduces JCPyV infection but not viral attachment or entry. (**A**) Cetirizine [200 μM] or DMSO was added to SVGA cells either 1 h prior to infection, concurrently with JCPyV (MOI = 1.5 FFU/cell), or at indicated times after infection and incubated at 37 °C for 48 h. Cells were then fixed and analyzed by ICW assay. Percent infection was determined by dividing the background subtracted signal from the 800 nm channel of each well with its corresponding 700 nm signal and normalizing to 100%. Graph represents triplicate samples in 3 biological replicates. (**B**) SVGA cells were pre-treated with cetirizine [200 μM] or DMSO at 37 °C for 1 h and then infected with JCPyV-647 on ice. Samples were fixed and analyzed by flow cytometry using a BD LSR II with an APC laser line (640 nm) to measure viral attachment. FACSDiva and FlowJo software was used to analyze data, and gating was performed to exclude dead or complex cells. At least 5000 events were analyzed per sample in 3 biological replicates. (**C**) SVGA cells were pre-treated with cetirizine [200 μM] or DMSO at 37 °C for 1 h and pre-chilled at 4 °C for 45 min. JCPyV-647 was added at 4 °C for 1 h, and plates were then shifted to 37 °C for 2 h before fixation. Cells were stained for pan-cadherin and DAPI and imaged on a Zeiss LSM 980 confocal microscope at 63× magnification. ROIs were drawn around the perimeter of each cell, excluding the plasma membrane using ImageJ, and the mean intensity values of internalized virus were quantified. At least 30 cells were analyzed per sample in 3 biological replicates. (**D**) Representative images from confocal microscopy. Nuclei (cyan), JCPyV-647 (magenta) and pan-cadherin (yellow). Scale bars = 20 μm. Error bars represent SEM, and statistical significance was determined using Student’s *t*-test. *, *p* < 0.05. n.s. = not statistically significant.

To further understand at which step in the JCPyV infectious cycle cetirizine reduced infection, a time of addition assay was performed. SVGA cells were treated with cetirizine at indicated time points prior to or after infection with JCPyV. Cetirizine significantly reduced infection at all evaluated time points, but the greatest reduction occurred between 1 h prior to and 6 h post-infection (hpi) (Figure 3A). These time points correlate with attachment, entry, trafficking, and GPCR signaling steps in the JCPyV infectious cycle [25,28,30]. The impact of cetirizine on viral attachment was evaluated using flow cytometry in which SVGA cells were treated with cetirizine or DMSO and then infected with Alexa 647-labeled JCPyV (JCPyV-647) on ice to allow for viral attachment. Analysis by flow cytometry revealed no significant difference in the mean fluorescence intensity between cetirizine and DMSO-treated samples, implying that cetirizine does not affect viral attachment to host cells (Figure 3B). The next step in the infectious cycle, entry, was assessed using confocal microscopy. SVGA cells pre-treated with cetirizine or DMSO were infected with JCPyV-647 at 4 °C for 1 h to allow for viral attachment and then shifted to 37 °C for 2 h for viral internalization. Cells were fixed and stained for pan-cadherin to mark the plasma membrane for imaging by confocal microscopy. Viral internalization was evaluated using ImageJ by measuring the mean fluorescence of internalized virus in DMSO- compared to cetirizine-treated cells. Results showed no reduction in viral internalization in cetirizine-treated samples compared to the control, indicating that cetirizine does not block viral entry (Figure 3C,D). In sum, cetirizine significantly reduced JCPyV infection in SVGA cells throughout the infectious cycle through a mechanism other than viral attachment and entry.

### 3.6. Paroxetine Reduces Viral Internalization in Glial Cells

**Figure 4:**
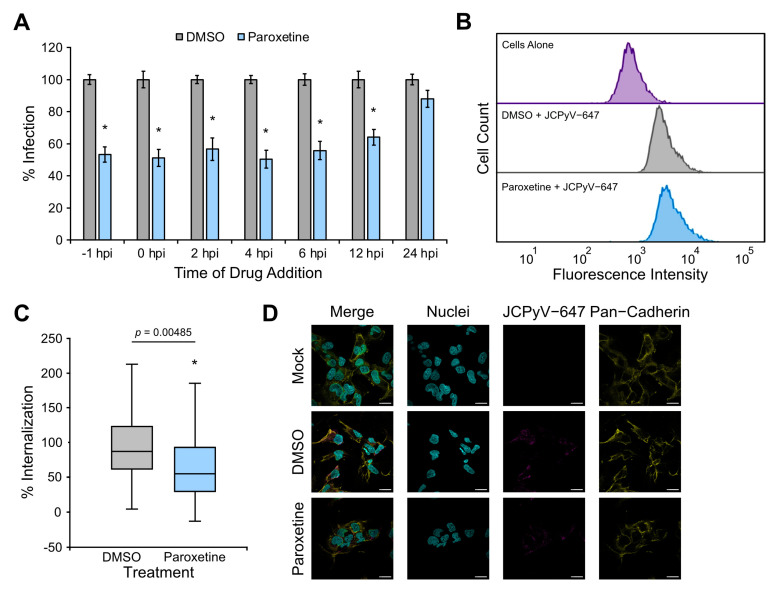
Paroxetine inhibits viral entry in glial cells. (**A**) Paroxetine [15 μM] or DMSO was added to SVGA cells either 1 h prior to infection, concurrently with JCPyV (MOI = 1.5 FFU/cell), or at indicated times post-infection and incubated at 37 °C for 48 h. Cells were then fixed and analyzed by ICW assay. The percent infection was determined by dividing the background subtracted signal from the 800 nm channel of each well with its corresponding 700 nm signal and normalizing to 100%. (**B**) SVGA cells were pre-treated with paroxetine [15 μM] or DMSO at 37 °C for 1 h and then infected with JCPyV-647 on ice. Samples were fixed and analyzed by flow cytometry using a BD LSR II with an APC laser line (640 nm) to measure viral attachment. FACSDiva and FlowJo software were used to analyze data, and gating was performed to exclude dead or complex cells. At least 5000 events were analyzed per sample. (**C**) SVGA cells were pre-treated with paroxetine [15 μM] or DMSO at 37 °C for 1 h and pre-chilled at 4 °C for 45 min. JCPyV-647 was added at 4 °C for 1 h, and plates were then shifted to 37 °C for 2 h before fixation. Cells were stained for pan-cadherin and DAPI and then imaged on a Zeiss LSM 980 confocal microscope at 63× magnification. Virus internalization was quantified using ImageJ. ROIs were drawn around the perimeter of each cell, excluding the plasma membrane, and mean intensity values of internalized virus were quantified. At least 30 cells were analyzed per sample. (**D**) Representative images from confocal microscopy. Nuclei (cyan), JCPyV-647 (magenta) and pan-cadherin (yellow). Scale bars = 20 μm. All graphs represent 3 biological replicates and error bars represent SEM. Statistical significance was determined using Student’s *t*-test. *, *p* < 0.05 or *p* value as indicated in figure.

To further explore which step in the JCPyV infectious cycle paroxetine acts upon, a time of addition assay was performed. SVGA cells were treated with paroxetine prior to, during, or post-infection with JCPyV at the indicated time points, and infection was quantified by ICW assay. Paroxetine significantly reduced infection at time points up to 12 hpi, and these early time points are consistent with viral attachment, entry, and trafficking as well as host GPCR signaling (Figure 4A) [25,28,30]. To investigate whether paroxetine impacts viral attachment to host cells, flow cytometry was utilized (Figure 4B). SVGA cells were treated with paroxetine or DMSO and incubated with JCPyV-647 on ice to allow viral attachment while preventing internalization. Flow cytometry analysis of the mean fluorescence intensity between samples treated with paroxetine or control showed no significant difference, indicating that paroxetine does not impact JCPyV attachment to SVGA cells (Figure 4B). Further, confocal microscopy was utilized to assess the effect of paroxetine on viral internalization (Figure 4C,D). SVGA cells were treated with paroxetine or DMSO, and experiments were performed as described above. The findings demonstrate that paroxetine significantly reduced viral internalization compared to the DMSO control by 32% in SVGA cells (Figure 4C). Overall, paroxetine inhibits the JCPyV infection of glial cells early in the infectious cycle, specifically during viral entry.

### 3.7. Paroxetine Treatment Alters Viral Entry and 5-HT_2C_ Receptor Properties in HEK Cells

**Figure 5:**
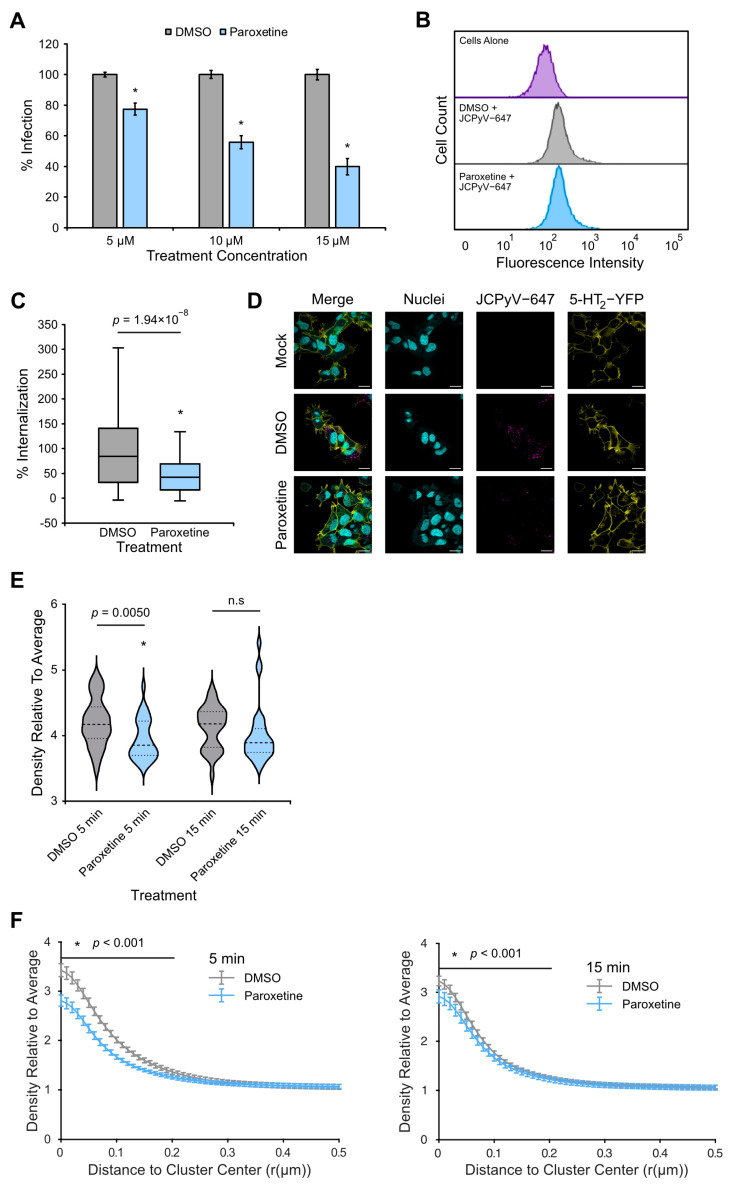
Paroxetine reduces JCPyV entry and receptor clustering in HEK cells. (**A**) HEK-2C-YFP cells were pre-treated with paroxetine or DMSO control at 37 °C for 1 h and infected with JCPyV (MOI = 1 FFU/cell) in the absence of drug at 37 °C for 1 h. Cells were fed with media containing paroxetine or DMSO and incubated at 37 °C for 48 h. After fixation, cells were stained for TAg and DAPI. FFU assay was used to quantify infection of 5 FOV/well at 20×, and percent infection was determined by dividing the number of TAg+ cells by the total number of cells and normalizing to 100%. Graphs represent 3 biological replicates performed in triplicate, and error bars represent SEM. (**B**) HEK-2C-YFP cells were pre-treated with paroxetine [15 μM] or DMSO at 37 °C and then infected with JCPyV-647 on ice. Samples were fixed and analyzed by flow cytometry using a BD LSR II with an APC laser line (640 nm) to measure attachment. FACSDiva and FlowJo software were used to analyze data, and gating was performed to exclude dead or complex cells. At least 5000 events were analyzed per sample. (**C**) HEK-2C-YFP cells were pre-treated with paroxetine [15 μM] or DMSO at 37 °C for 1 h and pre-chilled at 4 °C for 1 h. Cells were incubated with JCPyV-647 at 4 °C for 1 h (attachment), and plates were shifted to 37 °C for 2 h (entry) before fixation. Cells were stained with DAPI and imaged on a Zeiss LSM 980 confocal microscope at 63× magnification. Virus internalization was quantified using ImageJ. ROIs were drawn around the perimeter of each cell, excluding the plasma membrane, and mean intensity values of internalized virus were quantified. At least 30 cells were analyzed per sample. (**D**) Representative images from confocal microscopy. Nuclei (cyan), JCPyV-647 (magenta) and 5-HT_2_C-YFP (yellow). Scale bars = 20 μm. (**E**) HEK-2C-Dendra2 cells were pre-treated with paroxetine [15 μM] or DMSO at 37 °C for 1 h, pre-chilled at 4 °C for 1 h, and JCPyV-647 was added at 4 °C for 1 h. Media containing paroxetine or DMSO was added, and cells were incubated at 37 °C for 5 or 15 min before fixation. Cells were imaged on a custom FPALM microscope, and images were processed using custom MATLAB scripts. Clusters were identified by single linkage cluster analysis, and their density distributions are shown by violin plots. Violin plots represent an average of 30 cells. Dashed lines = means; dotted lines = quartile distributions. (**F**) Cluster properties were determined by RDF analysis, where an RDF value of 1 indicates local density equal to average, RDF > 1 indicates clustering, and RDF < 1 indicates depletion. The data shown in RDF graphs represent averages of a minimum of 5000 individual clusters. All graphs represent 3 biological replicates, and error bars represent SEM. Student’s *t*-test was used to determine statistical significance in (**A**–**C**) *, *p* < 0.05 or *p* value as indicated in figure. Wilcoxon signed-rank tests were used to determine statistical significance in (**E**,**F**) with a Bonferroni–Holm correction in (**F**) with *p* values indicated in figure. n.s. = not statistically significant.

To determine whether the 5-HT_2_ receptors were affected by paroxetine-specific reduction in JCPyV infection, HEK293A cells stably expressing 5-HT_2C_ receptors (HEK-2C-YFP cells) were treated with paroxetine or DMSO and infected with JCPyV. Paroxetine treatment resulted in a significant reduction in JCPyV infection in HEK-2C-YFP cells (Figure 5A). As in Figure 4, the impact of paroxetine treatment on viral attachment and entry was tested in HEK-2C-YFP cells by flow cytometry and confocal microscopy, respectively (Figure 5B–D). Paroxetine did not reduce viral attachment to cells (Figure 5B) but significantly reduced viral entry by 55% compared to DMSO-treated cells (Figure 5C,D), which was a greater reduction in entry than was observed in SVGA cells (Figure 4C,D).

Our previous research has shown that JCPyV induces the clustering of 5-HT_2_ receptors at 5 mpi, which is consistent with viral endocytosis [23,24]. To determine whether the paroxetine-induced reduction in JCPyV entry was attributable to alterations in receptor dynamics during entry, super-resolution microscopy techniques, FPALM and dSTORM, were utilized (Figure 5E,F). HEK293A cells stably expressing a photoactivatable 5-HT_2C_ receptor (HEK-2C-Dendra2 cells) were treated with paroxetine or DMSO, infected with JCPyV-647 at 4 °C for 1 h for viral attachment, and then shifted to 37 °C for viral internalization for 5 or 15 min before fixation and imaging. Analysis of the 5-HT_2_ cluster patterns in cells by single linkage cluster analysis revealed a highly significant decrease in average cluster density at 5 mpi (Figure 5E). Interestingly, a bimodal distribution of clusters was observed in the paroxetine-treated cells at 5 mpi but not 15 mpi (Figure 5E). Additionally, there was a significant decrease in cluster density across individual clusters as demonstrated by RDF analysis (Figure 5F). The RDF analysis shows that cluster density is decreased in paroxetine-treated cells up to 200 nm from the cluster center at 5 mpi. This cluster density difference between paroxetine and control is decreased at 15 mpi due to a decrease in receptor clustering in the DMSO-treated cells (Figure 5F). Importantly, the data were selected based on a cluster identification threshold of three times the average receptor density, suggesting paroxetine significantly reduces the density of the most dense clusters during JCPyV infection. These data suggest that paroxetine alters 5-HT_2_ receptor cluster properties at times consistent with viral entry.

### 3.8. Paroxetine Reduces β-Arrestin Recruitment and GPCR Signaling

**Figure 6:**
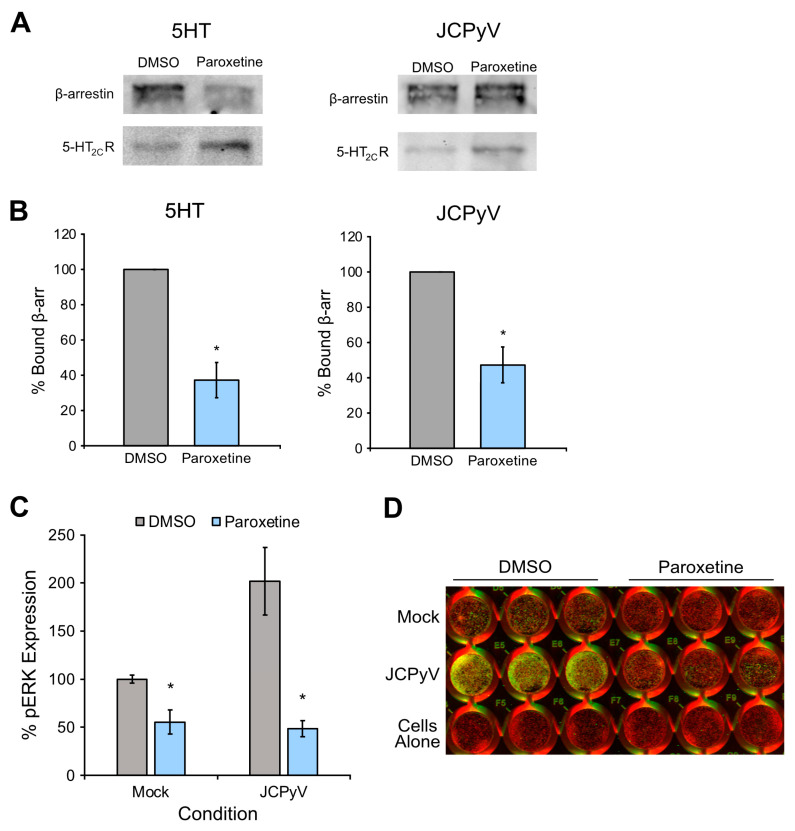
Paroxetine inhibits β-arrestin recruitment and ERK activation. (**A**) HEK-2C-YFP cells were pre-treated with paroxetine [15 μM] or DMSO at 37 °C for 1 h and then chilled to 4 °C for 45 min. Chilled cells were either infected with JCPyV (MOI = 3 FFU/mL) or stimulated with 5-HT [200 μM] at 4 °C for 1 h and then fed with pre-warmed media containing drug or DMSO at 37 °C for 15 min. Cells were removed from plates, lysed and incubated with GFP antibody conjugated magnetic agarose beads at RT for 2 h for immunoprecipitation of 5-HT_2C_R-YFP and bound β-arrestin. Samples were processed by SDS-PAGE and immunoblotted for β-arrestin and GFP (corresponding to 5-HT_2C_R-YFP). (**B**) Quantification of percent bound β-arrestin was determined using ImageJ by dividing the β-arrestin signal with the GFP signal and normalizing to 100%. Experiments were performed for 3 biological replicates. (**C**) Paroxetine [15 μM] or DMSO was added to SVGA cells at 37 °C for 1 h, and plates were then chilled at 4 °C for 15 min. Cells were either infected with JCPyV or mock infected at 4 °C for 1 h and fed back with pre-warmed media containing paroxetine or DMSO for 15 min. Cells were fixed, stained for pERK, and analyzed by ICW. % pERK expression was determined by dividing background-subtracted signal of the 800 nm channel in each well by the respective 700 nm signal and normalizing to 100%. Experiment was performed in triplicate for 3 biological replicates. Error bars represent SEM and statistical significance was determined using Student’s *t*-test. *, *p* < 0.05. (**D**) Representative image of one ICW replicate with triplicate samples quantified in (**C**).

In addition to being a serotonin reuptake transporter inhibitor, paroxetine also inhibits GRK2, which is a protein required for JCPyV infection presumably by priming the serotonin receptors for β-arrestin interaction with the 5-HT_2_Rs for viral entry [24]. To determine whether β-arrestin coupling to the 5-HT_2C_R is reduced by paroxetine treatment, GFP trap pulldown assays were performed [24]. HEK-2C cells expressing 5-HT_2C_R with a YFP tag were treated with paroxetine or DMSO control and either infected with JCPyV or stimulated with 5-HT on ice to activate receptor internalization events. Treated cells were incubated with pre-warmed drug- or DMSO-containing media, and cells were removed from plates, lysed, and incubated with magnetic beads conjugated with a GFP antibody. Samples were resolved by SDS-PAGE, and Western blot analysis was performed using antibodies to both 5-HT_2_R-YFP and β-arrestin (Figure 6A). Western blot quantification using ImageJ demonstrated a significant reduction in β-arrestin bound to 5-HT_2C_R in cells treated with paroxetine compared to DMSO treatment for both samples stimulated with 5-HT and samples infected with JCPyV (Figure 6B). Given that β-arrestin activation of the serotonin receptor activates the MAPK pathway, this result encouraged additional investigation into whether paroxetine also reduces MAPK signaling. Assays to measure pERK activation following infection were performed by treating SVGA cells with paroxetine or DMSO and infecting with JCPyV or media alone (mock). Cells were fixed and analyzed for pERK expression by ICW assay. A significant reduction in pERK activation was observed for both mock- and JCPyV-infected cells treated with paroxetine (Figure 6C). In sum, paroxetine significantly impaired β-arrestin recruitment to the 5-HT_2_R and reduced activation of the MAPK signaling pathway, which was possibly through the inhibition of GRK2.

## 4. Discussion

The lack of therapeutic options for treating JCPyV infection and PML highlights an opportunity for research to increase our understanding of viral infection and consequently identify novel therapeutic targets. To bridge existing knowledge gaps and discover potential antiviral therapies, this study validated seven FDA-approved, GPCR-associated hits identified in a high-throughput drug screen for reduction in JCPyV infection (Table 1) [34]. Upon further investigation two therapeutics, cetirizine and paroxetine were found to significantly reduce JCPyV infectivity in immortalized SVGA cells and were selected for further characterization of their antiviral properties (Figure 1). The majority of drugs that did not reliably reduce JCPyV infection were non-5-HT_2_R-related agonists or antagonists. This supports previously established knowledge that JCPyV utilizes 5-HT_2_Rs to mediate viral entry through β-arrestin-driven endocytosis [20,22]. Moreover, this study also suggests that other GPCRs, such as the histamine H1 receptor inhibited by cetirizine, may play a role in the JCPyV infectious cycle.

Cetirizine, a histamine H1 receptor antagonist currently used to relieve allergies [46], and paroxetine, a serotonin reuptake transport inhibitor and GRK2 inhibitor prescribed for depression [47], were further evaluated for their capacity to reduce JCPyV infection in primary cells. Compared to immortalized cells, primary cell lines provide a more clinically accurate human cell model void of variances in signaling pathways caused by immortalization. The treatment of NHA cells, a primary human astrocyte cell type, with cetirizine and paroxetine resulted in a significant reduction in JCPyV infection using both therapeutics (Figure 2A,C), indicating a conserved drug mechanism across immortalized and primary cell lines for both paroxetine and cetirizine. In RPTECs, a primary human kidney cell line, cetirizine and paroxetine also resulted in a significant reduction in viral infection (Figure 2B,D), suggesting that the mechanism of infection targeted by these drugs is conserved across both major cell types targeted by JCPyV in the host. Notably, the reduction in JCPyV infection in NHAs and RPTECs with paroxetine or cetirizine treatments yielded only minimal dose-dependent effects, which may be due to the narrow range of drugs tested and that higher doses were not included due to drug toxicity at higher concentrations.

Interestingly, cetirizine has also been shown to reduce the infectious ability of lentivirus pseudotyped with the SARS-CoV-2 spike protein [48] and was deemed a promising therapeutic approach in the management of COVID-19 during the recent pandemic [49]. Antihistamines are mainly developed for their use as allergy medications, but recent studies have revealed an increased applicability as anti-infective agents against viruses, bacteria, and parasites. Histamine receptor antagonists have been identified to reduce infection of several viruses such as SARS-CoV-2, filoviruses, and influenza virus through both in vitro and in vivo studies. The most common mechanism of action for these drugs involves targeting viral entry either by blocking receptor internalization or fusion events [50].

To characterize how cetirizine reduces JCPyV infection, the drug was applied at different time points throughout the infectious cycle, and a significant reduction in infection was observed at all evaluated time points. However, the greatest reduction occurred up to 6 h post-infection during the early stages of the infectious cycle (Figure 3A) [25,28]. Given the known function of cetirizine as a receptor antagonist, the impact of the drug on viral attachment and entry was explored using flow cytometry and confocal microscopy, respectively. Surprisingly, neither JCPyV attachment nor entry was reduced by treatment with cetirizine, suggesting that cetirizine is impacting a post-entry step in the infectious cycle (Figure 3B,C). Based on these findings, it is likely that cetirizine affects viral trafficking, signaling, or possibly viral transcription, yet the early timing of drug addition may have led to more significant impacts on infection in the time course experiment due to the length of treatment time (Figure 3A). Moreover, it is possible that the mechanism by which cetirizine reduces JCPyV infection is independent of its currently known function as a histamine receptor antagonist [46]. Additional exploration is necessary to further characterize how cetirizine inhibits viral infection.

Paroxetine is a serotonin reuptake transporter antagonist, and it is also an inhibitor of GRK2, which is an essential protein that mediates β-arrestin binding to the 5-HT_2_Rs [26,51,52]. Prior research from our lab has shown that GRK2 is required for JCPyV internalization and infection, and the knockdown of GRK2 using siRNA reduces β-arrestin coupling to the 5-HT_2_Rs [24]. β-arrestin and GRK involvement are required for the productive infection of many other viruses belonging to vastly diverse virus families [26]. Filoviruses, flaviviruses, and SARS-CoV-2 have all evolved to rely on signaling emanating from these two proteins, making them promising therapeutic targets [26].

To define the antiviral mechanism of paroxetine, a time-of-addition assay was performed, and paroxetine was found to reduce infectivity early in the infectious cycle (0–12 hpi) (Figure 4A), mainly correlating to viral attachment and entry. Additional investigation by flow cytometry demonstrated that viral attachment was not affected by paroxetine treatment in either glial (SVGA) cells or kidney (HEK-2C) cells (Figure 4 and Figure 5). However, an analysis of viral entry by confocal microscopy revealed that paroxetine significantly impaired viral internalization in both cell types (Figure 4 and Figure 5), suggesting that reduced viral entry could be due to the altered receptor properties induced by paroxetine.

To further explore whether paroxetine treatment affects 5-HT_2_R properties, receptor analysis upon infection was performed using super-resolution microscopy FPALM. Previous research from our lab using FPALM has shown that JCPyV induces serotonin receptor (5-HT_2_R) clustering at 5 mpi, which is consistent with clathrin-mediated endocytosis of the virus [23,24]. In this study, FPALM analysis demonstrated that 5-HT_2c_R clusters induced by JCPyV infection were decreased in cluster density and had altered cluster shapes in the presence of paroxetine at 5 mpi compared to the DMSO control (Figure 5E,F). Additionally, a bimodal distribution of cluster densities was observed in the paroxetine-treated cells at 5 mpi (Figure 5E), suggesting paroxetine may not fully inhibit receptor desensitization but rather reduce the number and size of clusters that could facilitate entry, reducing the chance of JCPyV internalization. This is further supported by the RDF analysis showing paroxetine reduces receptor density in clusters at 5 mpi up to 200 nm from the cluster center but does not entirely eliminate receptor clustering. However, this reduction occurs in the most dense clusters based on the thresholding used to identify clusters, suggesting the effects of paroxetine are particularly potent in reducing potential sites of viral internalization. Moreover, at 15 mpi, the cluster density in DMSO-treated cells is reduced in comparison to DMSO-treated cells at 5 mpi, suggesting that while the receptor is still clustering, the clusters are less dense at later times. This suggests that 5-HT_2C_R clusters at 5 mpi in response to JCPyV infection, and paroxetine treatment reduces the density of clusters, resulting in a decreased internalization of viral particles. Taken together, these data are consistent with our previous work and demonstrate that early receptor clustering may play a crucial role in viral endocytosis, which is reduced by paroxetine treatment. As a GRK2 inhibitor, it was hypothesized that treatment with paroxetine would prevent the recruitment of β-arrestin to the receptor, which is critical for JCPyV-receptor mediated endocytosis and infection.

To determine whether paroxetine inhibits β-arrestin recruitment to the receptor, 5-HT_2_R pulldown assays were performed. The results demonstrated a sizable reduction in receptor-bound β-arrestin upon treatment with paroxetine (Figure 6B), supporting the idea that paroxetine impacts viral entry by inhibiting β-arrestin binding to the 5-HT_2_R likely by inhibiting GRK2. Utilizing GRK2 as a drug target has also shown to be an effective method against influenza virus infection. Paroxetine treatment reduced influenza virus infection during viral entry, as this virus relies on GRK, β-arrestin, and MAPK signaling for internalization and uncoating [26,51]. Similar to influenza virus, β-arrestin recruitment during JCPyV infection activates the MAPK signaling pathway, resulting in the phosphorylation of ERK, which is an event required for productive infection [30,31,53].

The impact of paroxetine treatment on pERK activation was characterized, and it was confirmed that the drug significantly reduced MAPK signaling during infection (Figure 6C). Decreased pERK expression likely results as a downstream effect of the lowered β-arrestin binding during viral entry caused by paroxetine treatment. However, it is also possible that the mere presence of JCPyV inside the host cell could activate the MAPK pathway independently of GRK2 and β-arrestin, and thus a decreased level of internalized virus in the presence of paroxetine could result in a subsequent reduction in pERK expression.

Although viral entry seems to be an important step affected by paroxetine, the reduction in internalization is considerably lower than the total amount of reduction observed during the initial validation assay. One explanation may be that paroxetine also inhibits other steps in the JCPyV infectious cycle, resulting in an additive effect. Alternatively, this could be due to JCPyV virions entering through alternative mechanisms such as a non-receptor-dependent process [54]. For this to be established, however, additional research of other potential pathways is warranted. Interestingly, paroxetine was identified in a NIH-CC drug screen as an inhibitor of coronavirus [55] mouse hepatitis virus (MHV) and was demonstrated to reduce MHV entry. In addition to paroxetine, several other antidepressants have demonstrated antiviral activity against Ebola virus and SARS-CoV-2 through mechanisms involving viral replication and interaction with viral glycoproteins or by exhibiting direct virucidal effects [56,57]. In a study where paroxetine reduced the viral RNA copy number of SARS-CoV-2 in vitro, a combination of inhibitory effects was observed, supporting the proposal that paroxetine can inhibit viral infection through multiple modes of action [56]. Antidepressants have also been revealed to have antiviral benefits in clinical studies where a significant association between the intake of these drugs and a decreased risk of both mortality and hospitalization was observed in patients with ongoing SARS-CoV-2 infection [56,58].

Altogether, the results from this study characterize receptor-associated inhibitors paroxetine and cetirizine as potential therapeutics to treat JCPyV infection and the resulting neurological disease PML. It is expected that the GPCR inhibitors in this study would interfere with receptor-dependent endocytosis, but not receptor-independent endocytosis [17], yet cetirizine did not reduce viral entry. Previous reports have identified the viral entry of JCPyV as a promising target for drug discovery, but despite reassuring results in vitro using drugs like mirtazapine, clinical trials presented with mixed results and limited benefit [16,33,59,60]. Both drugs validated in this report are currently FDA approved and could possibly be repurposed into antiviral therapies with limited side effects and toxicity. This process is a well-demonstrated approach and was recently utilized for the discovery of treatment options during the COVID-19 pandemic [61]. Paroxetine is known to cross the blood–brain barrier and could therefore be used to reduce infection both in the kidneys and the brain, whereas cetirizine has a diminished ability to cross the blood–brain barrier and would be limited to treatment in the kidneys [62,63]. Further, it would be of interest to determine the effect of combining paroxetine with cetirizine or perhaps combining these inhibitors with other FDA-approved antiviral agents that block a post-receptor entry step in the viral lifecycle. For example, brincidofovir, an FDA-approved drug for poxvirus, inhibits mouse polyomavirus (MuPyV) infection in vitro by blocking a post-transcriptional step in MuPyV infection and reduces viremia and infection in the kidney in vivo [64]. It is possible that combining brincidofovir with paroxetine or cetirizine might result in a more profound inhibition of JCPyV infection. Thus, further exploration of the clinical implications of cetirizine and paroxetine and possible combinations with antivirals is warranted.

## 5. Conclusions

FDA-approved, GPCR-associated inhibitors with a target receptor gene count above zero in SVGA cells were selected for the validation of their potential antiviral activity against JCPyV. Cetirizine and paroxetine reduced JCPyV infection in immortalized and primary brain and kidney cells. Further mechanistic investigations showed that paroxetine reduced viral entry and cetirizine reduced a post-entry step in the infectious cycle. Paroxetine specifically reduced viral internalization and pERK signaling, which was likely by inhibiting receptor cluster density and/or β-arrestin binding to the 5-HT_2_R. This work demonstrates the possibility of GPCR-associated inhibitors being repurposed and used as antiviral therapeutics against JCPyV infection.

## Figures and Tables

**Table 1:** Drugs selected for further validation. Seven drugs hits characterized as GPCR inhibitors in the high-throughput drug screen published by Bond et al. [34]. All selected drugs are FDA approved and have an average target gene count above zero. Average gene counts are based on RNASeq data published by Wilczek et al. [45].

Agonist/Antagonist	Function	Use	FDA Status	Gene Count
Cetirizine	Histamine H1 receptor antagonist	Allergy	Approved	1280.3
Paroxetine	Serotonin reuptake transporter inhibitor GRK2 inhibitor	Depression	Approved	872.9
Pergolide	Dopamine receptor agonist	Parkinson’s disease	Approved	16.2
Pilocarpine	Muscarinic receptor agonist	Dry mouth	Approved	12.6
Pindolol	β-adrenoreceptor antagonist	Hypertension	Approved	5.6
Rizatriptan	5-HT_1B_ receptor agonist	Migraines	Approved	72.9
Telmisartan	Angiotensin II type 1 receptor antagonist	Hypertension	Approved	114.3

**Table 2:** Toxicity of drug concentrations utilized in the study. Cell viability of cell types utilized in this study was assessed after drug treatment at indicated concentrations and time points. Drug concentrations resulting in <80% viability were deemed toxic and omitted from additional validation studies.

Agonist/Antagonist	Cell Type & Time Point	Percent Viability		
		≥100%	≥90%	≥80%
Cetirizine	SVGA 48 h		150 μM/175 μM/200 μM	
	RPTEC 72 h			150 μM/175 μM/200 μM
	NHA 48 h		150 μM/175 μM/200 μM	
Paroxetine	SVGA 48 h	5 μM		10 μM/15 μM
	RPTEC 72 h		5 μM/10 μM	15 μM
	NHA 48 h		5 μM/10 μM	15 μM
Pergolide	SVGA 48 h		100 μM/125 μM	
Pilocarpine	SVGA 48 h		75 μM/100 μM/125 μM	
Pindolol	SVGA 48 h	150 μM	175 μM/200 μM	
Rizatriptan	SVGA 48 h		100 μM/125 μM	
Telmisartan	SVGA 48 h		5 μM	10 μM

## Data Availability

No new data were created or analyzed in this study. Data sharing is not applicable to this article.
